# Cognitive function and social cognition in adolescents with bipolar disorder: comparison between manic episode and remission period

**DOI:** 10.1007/s00406-025-01987-0

**Published:** 2025-03-03

**Authors:** Celal Yeşilkaya, Sezen Alarslan, Mustafa Tuncturk, Cagatay Ermis, Serkan Turan, Gul Karacetin

**Affiliations:** 1 Department of Child and Adolescent Psychiatry, Konya Ereğli State Hospital, Konya, Turkey; 2 Department of Psychiatry and Psychology, Mayo Clinic, Minnesota, United States of America; 3 Department of Child and Adolescent Psychiatry, Sahlgrenska University Hospital, Queen Silvia Children’s Hospital, Gothenburg, Sweden; 4 Department of Child and Adolescent Psychiatry, Uludağ University School of Medicine, Bursa, Turkey; 5 Department of Child and Adolescent Psychiatry, University of Health Sciences, Bakirkoy Prof. Dr. Mazhar Osman Research and Training Hospital for Psychiatry, Neurology and Neurosurgery, Istanbul, Turkey

**Keywords:** Bipolar disorder, Neurocognition, Social cognition, Adolescent

## Abstract

**Background:**

We aimed to investigate the extent of cognitive impairments in early-onset bipolar disorder (EBD) during manic episode in comparison to remission period.

**Method:**

30 healthy controls (HC) and 95 patients with EBD, with manic episode (*n* = 55) and remission period (*n* = 40) were included. Additionally, 31 (%56.4) of 55 patients with manic episode were re-evaluated during remission. A comprehensive cognitive battery was implemented to asses verbal and visual learning/memory, attention, inhibition, problem-solving, working memory, processing speed, and verbal fluency skills and global cognitive factor was calculated to estimate overall cognitive ability. Theory of mind (ToM) was evaluated using the Reading the Mind in the Eyes and Faux-Pas tests.

**Findings:**

Individuals in patient groups and HC were matched for gender and education. Patients in remission had a significantly older mean age than the other groups. Antipsychotic dosage was also higher in cases with mania. Patients with manic episode had moderate impairments in processing speed (Cohen’s d: 0.51–0.78), attention (d: 0.57), inhibition (d: 0.56–0.63) and global cognitive function (d: 0.54) compared to patients in remission period. Individuals in remission period had poorer performance in verbal memory (d: 1.03–1.32), working memory (d: 0.88–1.13), ToM (d: 0.60–0.87), processing speed (d: 1.21–1.27), problem solving (d: 0.56–0.67), attention (d: 0.58), inhibition (d: 0.89-1.00) and visual memory (d: 1.28–1.37) in comparison with HC.

**Conclusion:**

Our findings indicated that impairments in social cognition, processing speed, inhibition, and attention were more prominent in the manic episode. Future studies should focus on pharmaco- and psychotherapeutic interventions aimed to treat neurocognitive impairments.

**Supplementary Information:**

The online version contains supplementary material available at 10.1007/s00406-025-01987-0.

## Introduction

Bipolar Disorder (BD) is a chronic and recurrent psychiatric disorder, characterized by fluctuations in mood and energy, impairments in mood, energy, reasoning affect, thought processes, psychomotor activity, and functioning [1, 2]. Early-onset BD (EBD) is defined as the onset of symptoms before the age of 18 [3].

There is a growing consensus in past studies about the presence of neurocognitive impairment in EBD, however, there are differences between studies regarding the domains of impairment. For example, some studies reported impairments in verbal learning and memory, working memory (WM), visual learning/memory, and attention [4, 5]. Similarly, a previous meta-analysis suggested that young individuals with BD performed poorly in global cognition (Hedge’s g: 0.78), attention (g: 0.41–0.48), verbal memory and learning (g: 0.89–0.90), and WM (g: 0.79–0.99) and visual memory (g: 0.78) during euthymic phase compared to healthy controls (HC) [5]. Likewise, a systematic review indicated that the most consistent results observed were in the verbal memory ​​deficits, WM, and the effect sizes of attention, visual memory, and verbal fluency deficits were moderate [6]. Additionally, another meta-analysis suggested moderate to large effect sizes in verbal learning and memory, processing speed, WM, and attention in individuals with EBD in comparison with early-onset schizophrenia; but not in verbal fluency and visual memory [3]. In contrast, there are some studies indicating no impairments in attention in both manic episode and euthymic phase [7], and no visual memory [8, 9], processing speed [5], and WM [10] deficits during remission period.

Social cognition is defined as the ability to perceive social and verbal cues about other people and oneself [11]. The ability to predict, interpret, and explain the emotions and thoughts of others is referred to as theory of mind (ToM) [12]. While a previous systematic review and meta-analysis indicated ToM deficits with large effect sizes in EBD [13], another previous study suggested that 30–60% of patients with EBD have significant social deficits, particularly during depressive episodes or periods of subsyndromal depressive symptoms [14]. It is important to define the extent of the social deficits in EBD, given the fact that studies suggested that impaired ToM skills during hospitalization may pose a risk of recurrence and persistent low social functioning even after a year of hospitalization [15, 16].

Mood state in EBD is an important factor that might have an effect on neurocognitive deficits. However, some studies indicated no correlation between mood state and neurocognition [6], furthermore, another review suggested that there was a significant difference between EBD and HC, but no difference between cases in the manic and euthymic phases [17]. Likewise, a previous study suggested there was no difference in any domain between individuals in mania and euthymia [18]. Social cognitive deficits during manic episodes have been less researched. For instance, a systematic review and meta-analysis suggested that there was no greater impairment in social cognition in patients with manic episode compared to the cases in remission period [13]. Additionally, a study indicated emotion identification deficits in both BD-I and BD-II, even after controlling for mood state [19]. In summary, it is important to determine mood state-related differences to distinguish whether these deficits decline when patients remitted.

There are few studies about neurocognitive and social functions in EBD in different mood states, which are often characterized by small sample sizes and heterogeneous inclusion criteria [7, 18]. To our knowledge, there is no previous study evaluating neurocognition and social cognition comparing mood states in hospitalized adolescents with manic episode as well as their euthymic phase and in comparison to euthymic patients in the outpatient clinic. Our study aims to address these gaps by comparing neurocognitive functioning during manic episode and remission in EBD. We hypothesized that ToM is significantly impaired during manic episode in comparison with remission, and secondly, executive functions (attention, inhibition, processing speed, working memory) exhibits greater impairment during manic episode. Finally, we anticipated that the most impaired domain is processing speed among all cognitive functions in patients with EBD during manic episode.

## Method

### Participants

The study included 30 HC subjects and 95 patients with EBD, with 55 in manic episode and 40 in remission period, as shown in Figure S1. Patients between the ages of 12 and 18 who met the diagnostic criteria for Bipolar-I Disorder or Bipolar-II Disorder according to DSM-5 as a result of the psychiatric evaluation performed on adolescents in a tertiary-care psychiatric inpatient and outpatient unit between September 2021 and January 2024 and agreed to participate in the study has been included. Cases who were admitted to the hospital with a manic episode in the last 2 months and who could comply with neurocognitive tests were included in the manic episode group. There was no predetermined time for the interviews due to case-by-case differences in the course of manic episodes. The neurocognitive assessment lasted approximately 2 h, so the ability of the individuals to continue with the clinical interviews was reviewed at each visit, and tests were implemented once they were able to do them. In the remission group, the patients who had been in remission and had not required any increase in drug dosage for at least 2 months according to DSM-5 complete remission criteria were included [20]. The HC group comprised individuals who volunteered through advertisements in the similar epidemiological environment. A semi-structured interview called “Kiddie Schedule for Affective Disorders and Schizophrenia for School-Age Children Present and Lifetime Version, DSM-5, Turkish Version (K-SADS-PL-DSM-5)” was implemented to both the adolescents and the families to review or rule out the present and the past psychiatric diagnoses [21]. Individuals with any neuropsychiatric diagnosis were excluded from the healthy control group. Benzodiazepine use was discontinued on the day of neurocognitive evaluation.

Exclusion criteria included a diagnosis of EBD due to another medical condition, mental retardation in the adolescent or caregiver, presence of autism spectrum disorder significantly hindering mutual communication, history of serious neurological disorder, history of head trauma resulting in loss of consciousness, and meeting the diagnosis of schizophrenia according to DSM-5 [20].

Written consent was obtained from all subjects and their caregivers, and the study was approved by the ethics committee.

### Procedures

General information about the adolescents was collected through sociodemographic data forms completed collaboratively by families and adolescents. Diagnoses were confirmed via semi-structured interviews, reaching a consensus among all authors. Clinical Global Impression Scale (CGI); Child General Assessment Scale (CGAS); Young Mania Rating Scale (YMRS), Child Depression Rating Scale (CDRS), and Premorbid Adjustment Scale (PAS) were used to evaluate the psychopathology and the symptom severity. PAS and general information subtests of Wechsler Intelligence Scale for Children- Revised (WISC-R) and Wechsler Adult Intelligence Scale (WAIS-R) were used to evaluate premorbid functioning.

Remission criteria were defined as a YMRS total score of less than 7 and a CDRS-R score of less than 28, in accordance with previous literature [22, 23]. Additionally, patients were required to be in remission and not necessitate an increase in drug dosage for at least two months. Neuropsychological and ToM tests were implemented on all patients by the researcher to evaluate verbal learning and memory, WM, verbal fluency, processing speed, inhibition, attention, visual learning and memory, and social cognition. The tests implemented were: *(i)* Rey Auditory Verbal Learning Test to evaluate verbal learning and memory -the verbal learning (trials 1 to 5), late recall (trial 7) and true recognition subscores-, *(ii)* Auditory Consonant Trigrams (ACT) and Digit Span tests to evaluate WM, *(iii)* Controlled Word Association Test (COWAT) to evaluate phonemic verbal fluency, *(iv)* animal names version of the categorical fluency test to evaluate semantic verbal fluency, *(v)* Digit-symbol subtest of WISC-R, Trail making test- A (TMT-A) and Stroop-word subtests to measure psychomotor speed; *(vi) S*troop word-color subtest and interference scores to evaluate inhibition capacity; *(vii)* TMT- B and WCST to evaluate problem-solving capacities of the individuals. While continuous performance task (CPT) was implemented to evaluate attention, immediate and late recall scores of the Wechsler Memory Scale-visual reproduction subtest were implemented to evaluate visual learning and memory. And finally, social cognition and ToM were evaluated through the reading the mind in the eye test (RMET) and the Faux-pas test [24, 25].

### Statistical analysis

The suitability of the data for normal distribution was determined by kurtosis and skewness values. Normally distributed continuous variables are presented with mean and standard deviation values. To analyze the TMT-A and ST- word-color naming results, which do not comply with the normal distribution, with parametric tests, these data were converted to normal distribution by logarithmic transformation. ANOVA analysis of variance across multiple groups was used on continuous variables related to sociodemographic characteristics and disease characteristics. The Bonferroni method was used to reduce the type-I error rate in post-hoc analyses.

We conducted a priori power analysis using G*Power v3.1.9.7 [26]. Based on a two-tailed test, medium effect size (d = 0.60) which based on a previous study [27], and alpha = 0.05, a total sample of 92 participants was required for 80% power. Post-hoc power analysis for repeated-measures ANOVA (alpha = 0.05, eta-square = 0.073, sample size = 31) yielded a power of 0.85.

To test the hypotheses of the study, ANCOVA models were implemented by controlling for age in the first model. CGI-S scores and chlorpromazine equivalent antipsychotic doses were highly correlated with group membership and illness state; thus, they were not included in covariates. In the second model, results were adjusted to age, education, sex and PAS scores.

To analyze 31 cases re-evaluations during the remission period, repeated measures ANOVA was used.

Similar to the previous literature, a model, in which all tests were collected into a single factor, was created to calculate global cognition scores, with approximately half of the entire variance explained, and the scores of the subjects who could not complete a test due to cognitive impairment were calculated as equivalent to the score of the lowest-performing subject who was able to complete the test [28]. Principal component analysis (PCA) allows factor scores of neurocognitive tests to be obtained. Factor loadings of neurocognitive tests on global cognition are demonstrated in Table S1. IBM SPSS (Statistical Package for Social Sciences; SPSS Inc., Chicago, IL) 26.0 program was used to implement the analyses. In the results of all analyses, a *p*-value of less than 0.05 was considered significant.

## Results

HC (*n* = 30) and EBD (*n* = 95), with manic episode (*n* = 55) and remission (*n* = 40) subgroups were included in this study. Demographic, illness, and treatment characteristics are shown in Table 1. The mean age of all participants was 16.7 ± 1.6 years at the time of cognitive assessment, and remission cases had a statistically significantly older mean age than the other two groups. Patients with manic episode had poorer premorbid functioning according to PAS scores and the patients had poorer scores in the information subtest of the WISC-R or WAIS-R. There was no difference in aripiprazole and mood stabilizer use, while quetiapine and risperidone use were more common in manic episode.

**Table 1:** Demographic, illness, and treatment characteristics of study groups

Variables	ME, *n* = 55	RP, *n* = 40	HC, *n* = 30	F/x²	*p*
Age*, y, M ± SD	16.5 ± 1.4	17.4 ± 1.2	16.0 ± 2.1	32.3	**0.002** ^**a**^
Sex, n (%)				1.4	0.493
Female	24 (43.6)	22 (55.0)	16 (53.3)		
Male	31 (56.4)	18 (45.0)	14 (46.7)		
Education, y, M ± SD	9.4 ± 1.9	10.5 ± 1.4	9.5 ± 1.8	4.6	**0.012** ^**b**^
Hand Dominance, n (%)				1.7	0.437
Right	52 (94.5)	37 (92.5)	26 (86.7)		
Left	3 (5.5)	3 (7.5)	4 (13.3)		
Maternal education, y, M ± SD	6.6 ± 4.4	5.6 ± 3.8	9.8 ± 4.3	8.6	**< 0.001** ^**c**^
Paternal education, y, M ± SD	8.1 ± 3.9	6.9 ± 3.0	10.8 ± 3.7	10.8	**< 0.001** ^**c**^
Smoking, n (%)	18 (32.7)	8 (20.0)	7 (23.3)	5.3	0.263
Alcohol, n (%)	8 (14.5)	4 (10.0)	4 (13.3)	0.4	0.803
Substance use, n (%)	8 (14.5)	1 (2.5)	0 (0.0)	8.1	**0.017**
Total PAS, M ± SD	7.7 ± 4.4	6.1 ± 3.3	4.8 ± 3.5	6.1	**0.003** ^**d**^
General Info.**, M ± SD	7.5 ± 2.8	7.9 ± 3.1	10.4 ± 2.9	10.3	**< 0.001** ^**c**^
Psychiatric Comorbidity, n(%)					
ADHD	22 (40.0)	14 (35.0)	-	x^2^ = 0.3	0.620
Substance Use Disorder	1 (1.8)	0 (0.0)	-	FET	1.000
YMRS	22.2 ± 10.5	1.3 ± 1.6	-	t = 14.6	**< 0.001**
CDRS	28.1 ± 9.0	21.0 ± 3.9	-	t = 5.0	**< 0.001**
CGI-S	5.1 ± 1.0	2.0 ± 0.2	-	t = 22.6	**< 0.001**
CGAS	33.3 ± 12.6	84.1 ± 7.5	-	t = 24.5	**< 0.001**
History of Physical Abuse, n(%)	10 (18.2)	2 (5.0)	-	x^2^ = 3.7	0.056
Treatment					
Chlorpromazine eq dose, mg/d, *M* ± SD	1118.0 ± 664.5	448.1 ± 487.3	-	t = 5.7	**< 0.001**
Quetiapine, n (%)	47 (85.5)	18 (45.0)	-	x^2^ = 17.5	**< 0.001**
Risperidone, n (%)	44 (80.0)	20 (50.0)	-	x^2^ = 9.5	**0.002**
Haloperidole, n (%)	8 (14.5)	0 (0.0)	-	FET	**0.019**
Aripiprazole, n (%)	6 (10.9)	9 (22.5)	-	x^2^ = 2.3	0.126
Lithium	15 (27.3)	7 (17.5)	-	x^2^ = 1.2	0.265
Sodium Valproate	10 (18.2)	8 (20.0)	-	x^2^ = 0.1	0.823

*Age at the time of the assessment. ** Standardized score of WISC-R/WAIS information subtest. ^a^ RP > ME = HC; ^b^ RP > ME, RP = HC, ME = HC; ^c^ HC > ME = RP; ^d^ ME = RP, RP = HC, ME > HC. ADHD: attention-deficit hyperactivity disorder, FET: Fisher’s Exact Test, M: mean, PAS: premorbid adjustment scale, SD = standard deviation, x^2^: Chi-square test, y: years

As expected, there were significant differences between the manic episode and remission groups on YMRS, CDRS, CGI-S, and CGAS scores. Table 2 demonstrates the neurocognitive test results of case groups and HC. There were statistically significant differences between groups in all tests for verbal learning and memory, WM, and ToM, processing speed, attention, inhibition, and visual memory, except COWAT.

**Table 2:** Comparison of neurocognitive test results of case groups and healthy controls

				Model 1^***^						Model 2^****^						
Neurocognitive Tests	ME, *n* = 55	RP, *n* = 40	HC, *n* = 30	F	*p*	Cohen’s d*				F	*p*	Cohen’s d*				
**Verbal domain**						ME-RP**	ME-HC**	RP-HC**				ME-RP**	ME-HC**			RP-HC**
RAVLT																
Learning (1–5)	37.0 ± 9.2	39.5 ± 8.9	50.6 ± 7.9	24.2	**< 0.001**	-	1.59	1.32		18.9	**< 0.001**	-		1.59	1.32	
Late Recall (7)	5.9 ± 2.7	6.8 ± 2.4	10.1 ± 2.4	26.0	**< 0.001**	-	1.64	1.38		22.1	**< 0.001**	-		1.64	1.38	
True Recognition	9.8 ± 3.3	10.4 ± 3.6	13.5 ± 2.3	12.6	**< 0.001**	-	1.30	1.03		12.3	**< 0.001**	-		1.30	1.03	
**Working Memory**																
ACT	23.6 ± 7.4	24.8 ± 7.3	32.6 ± 6.5	17.7	**< 0.001**	-	1.29	1.13		13.6	**< 0.001**	-		1.29	1.13	
Digit Span																
Forward	5.7 ± 1.9	5.4 ± 1.9	7.0 ± 2.0	7.5	**0.001**	-	0.67	0.82		7.5	**0.003**	-		-	0.82	
Backward	3.9 ± 1.6	4.1 ± 2.0	5.9 ± 2.1	11.5	**< 0.001**	-	1.07	0.88		8.8	**< 0.001**	-		1.07	0.88	
**ToM**																
RMET	18.2 ± 3.7	18.8 ± 3.9	20.8 ± 2.6	8.5	**< 0.001**	-	0.81	0.60		6.3	**0.003**	-		0.81	0.60	
Faux-pas	27.7 ± 7.0	28.8 ± 6.2	33.9 ± 5.4	9.5	**< 0.001**	-	0.99	0.87		6.9	**0.001**	-		0.99	0.87	
**Verbal Fluency**																
COWAT	30.2 ± 11.8	32.1 ± 13.1	35.9 ± 10.5	2.8	0.067	-	-	-		1.5	0.220	-		-	-	
Semantic fluency	18.0 ± 6.0	17.7 ± 5.4	23.2 ± 4.9	10.4	**< 0.001**	-	0.95	1.07		7.5	**0.001**	-		0.95	1.07	
**Processing Speed**																
Digit-symbol	40.0 ± 16.2	50.1 ± 17.3	69.3 ± 14.2	33.0	**< 0.001**	0.60	1.92	1.21		27.3	**< 0.001**	-		1.92	1.21	
TMT-A^a, b^	1.7 ± 0.2	1.6 ± 0.2	1.4 ± 0.1	19.8	**< 0.001**	-	1.90	1.27		14.6	**< 0.001**	-		1.90	1.27	
Stroop word^a^	33.4 ± 11.3	28.1 ± 7.1	26.9 ± 5.3	6.9	**0.001**	0.56	0.74	-		4.2	**0.018**	-		0.74	-	
**Problem Solving**																
TMT- B^a^	196.3 ± 89.3^c^	141.6 ± 73.0^c^	71.5 ± 27.4	28.0	**< 0.001**	0.67	1.89	1.27		22.7	**< 0.001**	0.67		1.89	1.27	
WCST																
N^o^ of Categories	1.7 ± 2.0	3.7 ± 3.0^d^	5.5 ± 2.3	24.9	**< 0.001**	0.78	1.76	0.67		18.6	**< 0.001**	0.78		1.76	0.67	
Total error	70.0 ± 23.3	56.6 ± 26.5	38.4 ± 17.7	18.0	**< 0.001**	0.54	1.52	0.81		13.4	**< 0.001**	-		1.52	0.81	
Per. Responses	35.0 ± 24.2	33.1 ± 25.2	22.0 ± 11.8	3.0	0.052	-	0.68	-		2.1	0.124	-		-	-	
Per. errors	31.3 ± 18.6	29.7 ± 20.1	19.1 ± 9.3	4.7	**0.011**	-	0.82	-		4.7	**0.031**	-		0.82	-	
**Inhibition**																
Stroop-color word^a, b^	2.0 ± 0.2	1.9 ± 0.1	1.8 ± 0.1	21.9	**< 0.001**	0.63	1.26	1.00		16.4	**< 0.001**	-		1.26	1.00	
Stroop Interference	80.6 ± 45.7	57.8 ± 24.8	39.2 ± 16.0	16.5	**< 0.001**	0.62	1.21	0.89		12.9	**< 0.001**	-		1.21	0.89	
**Attention**																
CPT-Accuracy^d^																
Target Accuracy, %	84.1 ± 12.7	90.3 ± 12.3	95.7 ± 4.7	11.4	**< 0.001**	-	1.21	-		6.7	**0.002**	-		1.21	-	
Foil Accuracy, %	43.3 ± 18.8	49.9 ± 22.5	56.2 ± 21.0	4.1	**0.018**	-	0.65	-		3.2	**0.044**	-		0.65	-	
**Visual domain**																
Visual Reproduction																
Immediate Recall	28.4 ± 8.6	30.2 ± 8.3	38.8 ± 3.4	17.9	**< 0.001**	-	1.59	1.37		13.2	**< 0.001**	-		1.59	1.37	
Late Recall	19.0 ± 12.2	21.2 ± 12.2	34.0 ± 7.1	18.7	**< 0.001**	-	1.50	1.28		14.3	**< 0.001**	-				
**Global Cognition (Z)**	-2.5 ± 1.3	-1.8 ± 1.3	0.0 ± 1.0	40.0	**< 0.001**	-	2.16	0.54		33.6	**< 0.001**	-		2.16	0.54	

ACT: auditory consonant trigrams, COWAT: controlled oral word association test, CPT: continuous performance task, HC: healthy control, M: median, ME: manic episode, SD: standard deviation, Per.: Perseverative, RAVLT: rey auditory verbal learning test, RP: remission period, RMET: reading mind in the eye test, TMT: Trail Making Test, ToM: Theory of Mind, WCST: Wisconsin card sorting test. ^a^ Durations as second. ^b^ Logaritmic transformation. ^c^ ME (*n* = 53), RP (*n* = 39): 3 cases could not complete the test because they did not know the alphabet. ^d^ RP (*n* = 38): Due to computer malfunction, test data of the cases could not be entered. *Calculated as Cohen’s d. 0.2 corresponds to small, 0.5 medium, and > 0.8 large effect size. **The second group was taken as the reference category, and the effect sizes indicate the deterioration in the first group. ^***^ Adjusted for age. ^****^ Adjusted for age, sex, education, and PAS scores

The ANCOVA analyses and the effect sizes of case and control groups with adjustments for age (model 1) and age, education, sex, and PAS scores (model 2) are shown in Table 2. According to the model 1, cases with manic episode had poorer performance than the HC group in all tests based on measuring neurocognition and social cognition; they also had worse results than the patients in remission period in the Digit symbol (Cohen’s d: 0.60), TMT-B (d: 0.67), TMT B-A (d: 0.68), Stroop word (d:0.56), color word (d: 0.63) and interference (d: 0.62), WCST completed categories (d:0.78), correct responses (d: 0.51), total errors (d: 0.54) sub-scores and CPT target accuracy (d: 0.50) and omission error (d: 0.50) sub-scores. Patients in the remission period had poor performance with varying effect sizes in all tests except COWAT, Stroop word, and foil accuracy sub-scoring of CPT in comparison with HC. Although the significant difference between both case groups and HC remained significant, the difference between ME and RG did not reach statistical significance in Model 2.

The neurocognitive test results, which were implemented to 31 cases during their manic episode and remission periods, are summarized in Table 3. Both RMET and Faux-pas test results were significantly poorer during manic episode. Participants had better performance during remission period compared to their manic episode in terms of TMT subtests. In the CPT, target accuracy and omission error parameters were better during remission phases of the cases than during manic episode. Patients had better scores during remission period in both visual reproduction scores in comparison with their scores during manic episode.

**Table 3:** Changes in neurocognitive test results of case groups in repeated measurements depending on the episode of the disorder

Neurocognitive Test Scores	Mania (*n* = 31)	Remission (*n* = 31)	F	*p*
**Verbal domain**				
RAVLT				
Learning (1–5)	38.2 ± 8.9	39.1 ± 9.2	0.5	0.506
Late Recall (7)	5.9 ± 3.1	6.3 ± 2.7	0.1	0.715
True Recognition	10.03 ± 3.2	10.23 ± 2.7	0.1	0.743
**Working Memory**				
ACT	24.0 ± 7.7	25.6 ± 8.3	2.4	0.134
Digit Span				
Forward	5.7 ± 2.1	5.8 ± 2.3	0.0	0.905
Backward	4.2 ± 1.9	4.6 ± 1.7	1.6	0.219
**ToM**				
RMET	18.7 ± 3.6	20.1 ± 3.7	5.3	**0.028**
Faux-pas	28.7 ± 6.3	31.8 ± 6.1	6.8	**0.014**
**Verbal Fluency**				
COWAT	31.8 ± 12.9	29.9 ± 13.8	1.6	0.223
Semantic fluency	17.81 ± 6.5	17.16 ± 5.1	0.3	0.566
**Processing Speed**				
Digit-symbol	41.1 ± 13.8	47.9 ± 12.4	6.3	0.018
TMT-A^a, b^	1.7 ± 0.2	1.5 ± 0.2	13.6	**0.001**
Stroop word^a^	42 ± 55.7	30.3 ± 9.3	1.5	0.226
**Problem Solving**				
TMT- B^a^	195.8 ± 93.6	145.9 ± 80.2	7.5	**0.011**
WCST				
N^o^ of Categories	1.9 ± 2.3	2.6 ± 2.2	3.5	0.070
Total error	69.3 ± 25.7	57.8 ± 20.4	8.1	**0.008**
Per. Responses	37.6 ± 29.6	41.0 ± 29.5	0.4	0.518
Per. errors	33.5 ± 22.4	34.5 ± 22.4	0.1	0.798
**Inhibition**				
Stroop-color word^a, b^	2.0 ± 0.2	1.9 ± 0.1	16.8	**< 0.001**
Stroop Interference	66.8 ± 64.5	50.3 ± 16.0	2.1	0.160
**Attention**				
CPT-Accuracy^d^				
Target Accuracy, %	84.7 ± 14.9	91.0 ± 10.1	11.0	**0.002**
Foil Accuracy, %	47.3 ± 19.2	49.0 ± 20.9	0.1	0.722
**Visual domain**				
Visual Reproduction				
Immediate Recall	29.6 ± 7.2	31.6 ± 8.2	4.7	**0.039**
Late Recall	19.6 ± 11.0	24.6 ± 12.9	8.1	**0.008**

ACT: auditory consonant trigrams, COWAT: controlled oral word association test, CPT: continuous performance task, HC: healthy control, M: median, ME: manic episode, SD: standard deviation, Per.: Perseverative, RAVLT: rey auditory verbal learning test, RP: remission period, RMET: reading mind in the eye test, TMT: Trail Making Test, ToM: Theory of Mind, WCST: Wisconsin card sorting test. ^a^ Durations as second. ^b^ Logaritmic transformation

## Discussion

To the best of our knowledge, this is the first study that investigated neurocognitive and social cognitive impairments in individuals with EBD in different mood states in follow-up. Individuals in the manic episode had significant impairments in all neurocognitive domains, while all domains except verbal fluency were impaired in patients in remission period compared to HC, aligning with the previous research which suggested that there are deficits in attention, working memory, and inhibition domains in euthymic cases with EBD compared to HC [29]. Impairments in attention, response inhibiton, problem-solving and processing speed were more pronounced during manic phase. ToM deficits were present in both case groups compared to HC, with greater effect sizes in patients in manic episode, but no significant difference was observed between individuals in manic episode and remission period. Patients had lower performance in ToM tests during manic episode compared to their remission period. Processing speed deficits had the largest effect size among other domains in manic episode compared to HC.

The findings of our study align with the previous literature that suggested no relationship between ToM deficits and mood state [13]. The continuation of the impairment even in euthymia might indicate that these deficits might be a trait of disorder, rather than state-marker, as accumulating research in adult BD suggested repeatedly [30–32].

In contrast with the previous literature [6–8, 33], the impairments in processing speed, attention, inhibition and attention were more pronounced during manic phase and can be both trait- and state-marker of the illness. These domains were closely related to prefrontal cortex, which can be a indication of impairments in noradrenergic/dopaminergic imbalance during mania [34, 35]. In line with the previous literature [8, 36], one of most impaired neurocognitive skill during manic episode was processing speed. One possible explanation of our findings can be overall severity of the illness at our center as the study center serves as tertiary-care psychiatric inpatient and outpatient unit for severe cases across the country where most severe cases referred for inpatient treatment [37], as seen in high antipsychotic doses during mania, likewise, previous research indicated that processing speed is related to the medication effects [34]. Therefore, this findings should be replicated in independent samples with hipomanic or less severe manic episodes.

Cognitive impairments in EBD, which are more evident during the manic episode (e.g. in ToM, processing speed, problem solving and attention) recently began to be targeted for both pharmacological and psychological treatments [38, 39]. Cognitive remediation was reported to have positive effects on cognitive skills in children with ADHD and adults with BD, and a recent review suggested that it may be effective in EBD [38]. Another recent study indicated that Cogmed Working Memory Training™ Program has promising positive effects on working memory deficits in EBD [40]. These interventions in EBD may contribute to prevent these impairments with early intervention and to increase functioning.

Some methodological limitations should also be considered to interpret our findings. First, single-center design of the study results in a limited sample group. Second, various treatment strategies can affect neurocognitive profiles of the participants. Also, sample size was modest to provide adequate statisical power for milder differences between case groups. This limitation also hindered to reach adequate power in follow-up visits and reduces the robustness of our results in sensitivity analysis when further covariates were added to the model. On the other hand, the underrepresentation of bipolar-II disorder in our sample was a key limitation, as our unit serves as a referral center for manic episode cases. Thus, we were not able to stratify our results with respect to bipolar subtypes. Finally, longer follow-up period is needed to be observed to distinguish long term consequences.

However, mixed-design study enabled within-individual design, comparing different illness states in the same samples. Also, a comprehensive neuropsychological battery was implemented to cover various domains of cognition. Thus, our study investigated neurocognitive impairments in EBD with multiple aspects, however without suggesting solutions for mentioned deficits.

## Conclusion

Individuals with EBD had significant impairments in ToM, processing speed, inhibition, problem solving, attention, and visual memory domains during mania. WM deficits are present in EBD regardless of mood state. Early interventions to these specific impairments in neuro- and social cognition may contribute to improve functioning. Further studies investigating specific pharmaco- and psychotherapeutic interventions targeting neurocognitive deficits are needed.

## Electronic supplementary material

Below is the link to the electronic supplementary material.

Supplementary Material 1

## Data Availability

Data available on request from the authors.
