# Supplementary material for: Cognitive function and social cognition in adolescents with bipolar disorder: comparison between manic episode and remission period
Source: Eur Arch Psychiatry Clin Neurosci. 2025 Mar 3;276(2):841–9. doi: 10.1007/s00406-025-01987-0 (PMC12953281; doi:10.1007/s00406-025-01987-0)
Supplement: Supplementary file 1 — Supplementary Material 1 [file 406_2025_1987_MOESM1_ESM.docx]

**
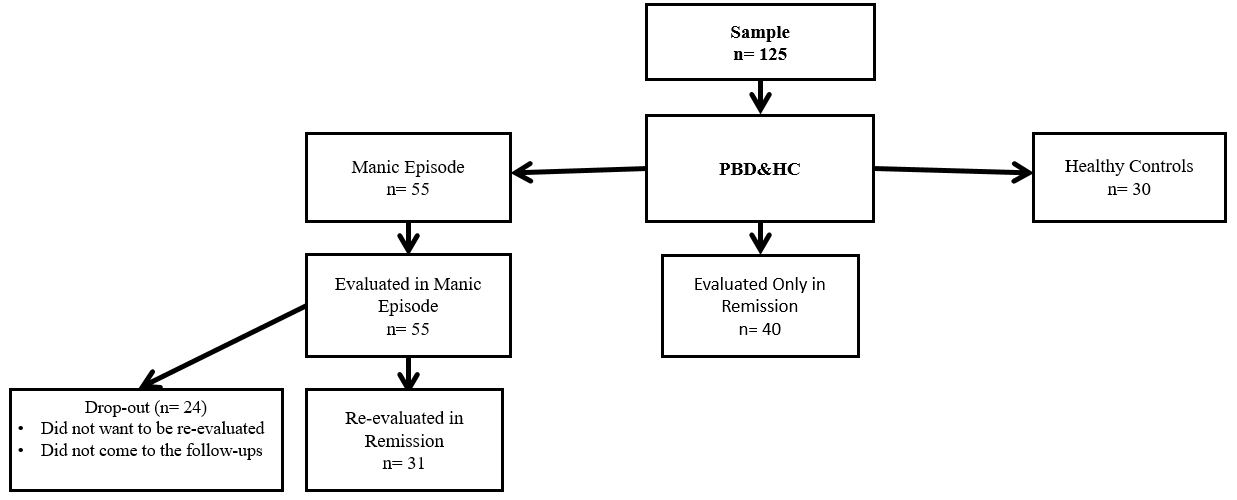
**

Figure S1: Summary of Main- and Subgroups

**Table S1.** Factor Loadings of Neurocognitive Tests on Global Cognition

| Pattern Matrix | Global Cognition |
| --- | --- |
| Digit-symbol Test (WISC-R) | 0.879 |
| Rey Learning | 0.851 |
| Visual Reproduction- Late Recall | 0.838 |
| Wisconsin Card Sorting Test-Correct Answer | 0.821 |
| Visual Reproduction- Immediate Recall | 0.802 |
| Trail making test- A (Log) | -0.785 |
| Auditory Consonant Trigrams | 0.742 |
| Stroop Interference | -0.657 |
| Digit Span Test- Backward | 0.638 |
| Continuous Performance Task- Target Accuracy | 0.587 |
| Wisconsin Card Sorting Test- Perseverated Error | -0.478 |

Global Cognitive Factor explaines approximately half of the explained variance
